# SNARE Regulatory Proteins in Synaptic Vesicle Fusion and Recycling

**DOI:** 10.3389/fnmol.2021.733138

**Published:** 2021-08-06

**Authors:** Chad W. Sauvola, J. Troy Littleton

**Affiliations:** ^1^The Picower Institute for Learning and Memory, Department of Brain and Cognitive Sciences, Massachusetts Institute of Technology, Cambridge, MA, United States; ^2^Department of Biology, Massachusetts Institute of Technology, Cambridge, MA, United States

**Keywords:** SNARE, synapse, synaptic vesicle, neurotransmitter release, membrane trafficking, *Drosophila melanogaster*, *Caenorhabditis elegans*

## Abstract

Membrane fusion is a universal feature of eukaryotic protein trafficking and is mediated by the soluble *N*-ethylmaleimide sensitive factor attachment protein receptor (SNARE) family. SNARE proteins embedded in opposing membranes spontaneously assemble to drive membrane fusion and cargo exchange *in vitro*. Evolution has generated a diverse complement of SNARE regulatory proteins (SRPs) that ensure membrane fusion occurs at the right time and place *in vivo*. While a core set of SNAREs and SRPs are common to all eukaryotic cells, a specialized set of SRPs within neurons confer additional regulation to synaptic vesicle (SV) fusion. Neuronal communication is characterized by precise spatial and temporal control of SNARE dynamics within presynaptic subdomains specialized for neurotransmitter release. Action potential-elicited Ca^2+^ influx at these release sites triggers zippering of SNAREs embedded in the SV and plasma membrane to drive bilayer fusion and release of neurotransmitters that activate downstream targets. Here we discuss current models for how SRPs regulate SNARE dynamics and presynaptic output, emphasizing invertebrate genetic findings that advanced our understanding of SRP regulation of SV cycling.

## Introduction

Eukaryotes rely on membrane-bound organelles to organize and transport material between cellular compartments (Wickner and Schekman, 2008). Transport between membrane-bound compartments and secretion of cellular cargo requires fusion of opposing lipid bilayers (Rothman, 1994; Jahn and Südhof, 1999). A large family of membrane associated SNARE proteins constitute the minimal molecular machinery required for membrane fusion by assembling into energetically favorable coiled-coil bundles that pull opposing lipid bilayers together to induce fusion (Söllner et al., 1993; Jahn and Scheller, 2006; Südhof and Rothman, 2009). Most cargo do not require a trigger for release and are trafficked into secretory vesicles destined for immediate fusion with the plasma membrane via the constitutive secretory pathway (Burgess and Kelly, 1987). Many cells including neurons also display a regulated secretion pathway for fast stimulus-dependent cargo release, typically in response to transient rises in intracellular Ca^2+^. Regulated secretion is mediated by a large cohort of SNARE regulatory proteins (SRPs) that control the timing and localization of SNARE assembly (Südhof and Rothman, 2009). Although some SRPs like *N*-ethylmaleimide sensitive factor (NSF), the soluble NSF attachment proteins (SNAPs) and Unc18 function in both constitutive and regulated secretion, others like Unc13, Complexin (Cpx), Synaptotagmin 1 (Syt1), Rab3-interacting molecule (RIM), and Tomosyn (Tom) provide unique temporal and spatial control of regulated secretion.

Many SRPs are present in all eukaryotes, suggesting they existed in the last common ancestor (Bennett and Scheller, 1993; Littleton, 2000; Lloyd et al., 2000; Varoqueaux and Fasshauer, 2017; Göhde et al., 2021). Others appeared later in multi-cellular eukaryotes that required more extensive cell-cell communication (Barber et al., 2009; Ryan and Grant, 2009). Gene duplication events occurring in vertebrate lineages generated orthologs of most SRPs in chordates. This redundancy is often absent in non-vertebrate lineages, facilitating genetic analysis of conserved membrane trafficking mechanisms in simpler model eukaryotes like the budding yeast *Saccharomyces cerevisiae*, the nematode *Caenorhabditis elegans* and the fruit fly *Drosophila melanogaster* (Novick et al., 1980; Bargmann, 1993; DiAntonio et al., 1993; Littleton and Bellen, 1995; Sato et al., 2014). Behavioral screens for temperature-sensitive (TS) paralytic mutants in Drosophila identified several conserved SRPs that contribute to SV release (Siddiqi and Benzer, 1976; Chen et al., 1991; van der Bliek and Meyerowitz, 1991; Kawasaki et al., 1998; Littleton et al., 1998, 2001b; Tolar and Pallanck, 1998; Kawasaki and Ordway, 1999; Rao et al., 2001; Babcock et al., 2004; Guan et al., 2005; Iyer et al., 2013). Similarly, screens for *C. elegans* mutants displaying motor paralysis, uncoordinated locomotion or altered sensitivity to the acetylcholinesterase inhibitor aldicarb have revealed key functions for multiple SRPs (Brenner, 1973; Hosono et al., 1992; Bargmann, 1993; Nguyen et al., 1995; Miller et al., 1996; Richmond et al., 1999; Sieburth et al., 2005). Given the conservation of SRPs across evolution, reverse genetic approaches have also been used to define functions for these proteins in Drosophila and nematodes (Schwarz, 1994; Richmond and Broadie, 2002; Harris and Littleton, 2015). The accessibility of peripheral neuromuscular junctions (NMJs) for electrophysiology and imaging has also facilitated characterization of SNARE and SRP function in SV cycling in Drosophila and *C. elegans* (Jan and Jan, 1976; Richmond and Jorgensen, 1999; Peled and Isacoff, 2011; Melom et al., 2013).

The SV cycle is initiated following action potential firing and depolarization of presynaptic terminals that cause transient voltage-gated Ca^2+^ channel opening (Katz, 1969; Sudhof, 2004). Subsequent spikes in local [Ca^2+^] trigger fusion of SVs that are docked and primed at specialized release sites known as active zones (AZs) (Zhai and Bellen, 2004; Ackermann et al., 2015; Ghelani and Sigrist, 2018). Following Ca^2+^ influx, SVs fuse at individual AZs in a probabilistic manner that is governed by a range of factors including local Ca^2+^ channel density and SV distance from the source of Ca^2+^ influx (Meinrenken et al., 2002; Bucurenciu et al., 2008; Böhme et al., 2016; Akbergenova et al., 2018; Neher and Brose, 2018). Release probability (*P*_*r*_) for SV fusion can be approximated by measuring AZ *P*_*r*_, which varies across neuronal subclasses and within the AZ population of a single neuron (Atwood and Karunanithi, 2002; Koester and Johnston, 2005; Peled and Isacoff, 2011; Holderith et al., 2012; Melom et al., 2013; Akbergenova et al., 2018; Neher and Brose, 2018; Karlocai et al., 2021). Most SVs are released via Ca^2+^-dependent evoked release, although some fuse in a stimulus-independent mode called spontaneous release. After fusion with the presynaptic plasma membrane, several endocytic routes for membrane and protein retrieval recover individual SVs, or larger membrane patches that traffic through endosomal compartments for further sorting (Soykan et al., 2016; Gan and Watanabe, 2018; Chanaday et al., 2019). Reformed SVs acidify through the action of the vesicular H^+^ pump, load neurotransmitters by vesicular H^+^ antiporters, and subsequently re-enter the SV pool for additional rounds of release (Sudhof, 2004).

In this review we examine current models for how SRPs guide SNAREs through their assembly/disassembly cycle, focusing on insights from invertebrate genetic studies of SV fusion. We also highlight biochemical approaches that guided reverse genetic experiments and provided context for interpreting genetic studies. The biochemistry and genetics of mammalian SV fusion have been described in prior reviews (Südhof, 2013; Neher and Brose, 2018; Rizo, 2018; Brunger et al., 2019). Key invertebrate and mammalian SRP phenotypes and their predicted molecular function are described in Table 1. This review begins with a description of the mechanism enabling SNAREs to overcome innate repulsion between opposing membranes, and the role of Unc13 and Unc18 in regulating SNARE availability for partial assembly. The SRPs Syt1 and Cpx then arrest SNARE assembly in a partially zippered state and subsequently promote Ca^2+^-dependent fusion. After fusion, NSF and SNAPs disassemble the SNARE complex to recharge individual SNARE proteins for further cycles of release. Intrinsic SNARE properties protect SNAREs from spontaneous reassembly post-fusion with help from the SRPs Unc18 and Tomosyn. Finally, RIM and Rab3 cooperate with Unc13 to re-position endocytosed SVs for subsequent docking and priming. Each of these steps provide avenues for modulation of SV release that can impact synaptic strength and plasticity.

**TABLE 1:** Summary of synaptic and behavioral phenotypes in *Mus musculus* (*M. mus*), *Caenorhabditis elegans* (*C. ele*), and *Drosophila melanogaster* (*D. mel*) SNARE and SRP mutants.

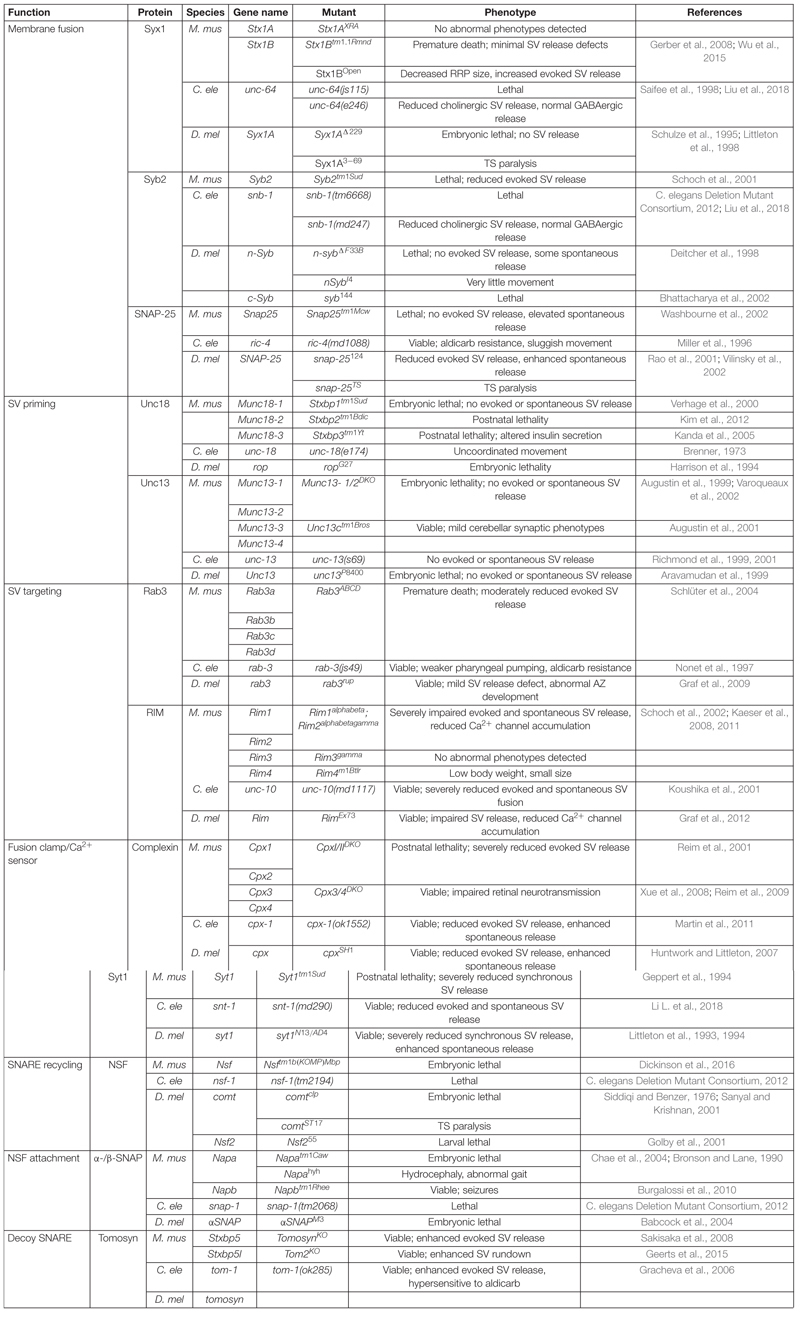

### SV Fusion Is Mediated by SNARE Complex Assembly

Lipids form stable bilayer membranes that innately repel each other through electrostatic forces and hydration repulsion (Milovanovic and Jahn, 2015; Robertson, 2018). Binding and assembly of SNARE proteins embedded in distinct bilayers is an energetically favored event that provides sufficient input to disrupt and fuse opposing membranes (Weber et al., 1998; McNew et al., 2000b; Tucker et al., 2004). SNAREs are a large protein family characterized by a ∼70 amino acid α-helical heptad repeat known as the SNARE motif. Based on their primary subcellular location, SNAREs are classified as vesicular (v-) or target (t-) membrane SNAREs. A secondary classification scheme defines the proteins as Q- or R-SNAREs depending on whether a glutamine (Q) or arginine (R) is encoded at a highly conserved central hydrophilic layer within the 16-layer SNARE coil. A fusion-competent SNARE complex is formed when three Q-SNARE helices combine with one R-SNARE helix of an opposing membrane (Weimbs et al., 1997; Fasshauer et al., 1998). Across species, the SNARE complex mediating SV fusion is composed of the v-SNARE Synaptobrevin 2 (Syb2, also known as vesicular associated membrane protein (VAMP)) and the t-SNAREs Syntaxin 1 (Syx1) and Synaptosomal associated protein of 25 kilodaltons (SNAP-25) (Söllner et al., 1993). Like all known SNARE complexes, the SV SNARE complex is composed of four α-helices, a Q-helix from Syx1, two Q-helices from SNAP-25 and one R-helix from Syb2. Syb2 and Syx1 are C-terminal anchored transmembrane proteins translated on cytosolic ribosomes and post-translationally inserted into membranes by the transmembrane recognition complex (TRC) (Trimble et al., 1988; Bennett et al., 1992b; Kutay et al., 1993; Borgese et al., 2003). SNAP-25 lacks a transmembrane domain and is post-translationally embedded in membranes via palmitoylation of a cysteine-rich central region (Oyler et al., 1989; Gonzalo and Linder, 1998).

**FIGURE 1:**
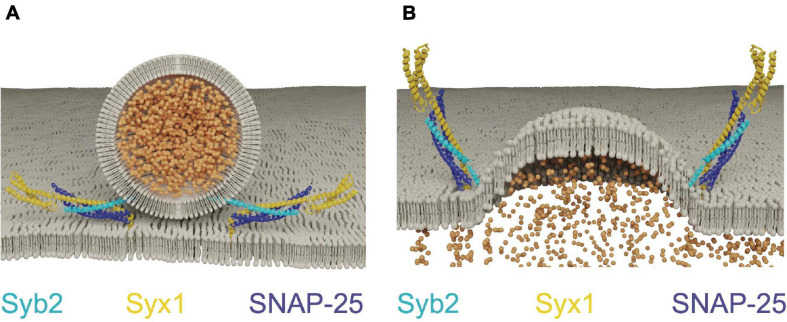
SNAREs assemble between opposing membranes to drive membrane fusion and neurotransmitter release. **(A)** One helix of the v-SNARE Syb2 assembles with three t-SNARE helices (one from Syx1 and two from SNAP-25) to form a four-helical *trans-*SNARE complex between opposing membranes. **(B)** Full SNARE zippering converts the *trans-*SNARE complex to a *cis*-SNARE complex to drive membrane fusion and neurotransmitter release. PDB structures in this and subsequent figures were obtained from the cited sources and rendered on membranes to highlight their role in SV fusion. Adapted from Fernandez et al. (1998); Sutton et al. (1998).

In their native state, SNAREs are disordered filaments that project from their carrier membranes into the cytosol (Fasshauer, 2003). Each protein displays selective binding to a set of cognate SNAREs that zipper together to form a highly structured four-helical SNARE bundle (Sutton et al., 1998; McNew et al., 2000b). Incorporation of individual SNARE filaments into the structured SNARE complex releases free energy that is harnessed to overcome the innate repulsion between opposing lipid membranes. Two competing models for the order of SNARE incorporation into the SNARE complex have been described (Rizo, 2018). One model proposes t-SNARE dimers of Syx1 and SNAP-25 are formed before the v-SNARE Syb2 is engaged. A more recent model argues the SRP Unc18 chaperones assembly of Syx1 and Syb2, ensuring proper alignment prior to SNAP-25 incorporation (Ma et al., 2013; Jiao et al., 2018). These two models converge once cognate SNARE recognition is established to form a partially zippered SNARE configuration known as the *trans-*SNARE complex, with transmembrane segments residing on separate compartments and full SNARE assembly being temporarily arrested (Figure 1A). SNARE zippering is directional, initiating at the free N-terminal end and progressing through the membrane embedded C-termini (Sutton et al., 1998; Pobbati et al., 2006; Stein et al., 2009; Hernandez et al., 2012). Full zippering through the C-terminus drives fusion by converting the *trans-*SNARE complex to a *cis*-complex where all transmembrane segments are embedded in the same bilayer (Figure 1B). The specific arrangement of SNARE complexes between fusing membranes and the number of complexes required for SV fusion remain unclear. However, current models suggest efficient fusion requires several SNARE complexes to be arranged like “spokes on a wheel” around the fusion pore formed between opposing membranes (Hua and Scheller, 2001; Kümmel et al., 2011; Shi et al., 2012).

Genetic analysis of SNARE mutants in Drosophila and *C. elegans* support an essential and conserved role for the SNARE complex in mediating SV fusion. In Drosophila, Syx1 is essential for fusion of both SVs and post-Golgi vesicles with the plasma membrane (Broadie et al., 1995; Schulze et al., 1995; Schulze and Bellen, 1996; Burgess et al., 1997). This dual function has made it difficult to define the precise role of Syx1 in SV release, as complete absence of the protein prevents cell viability. *Syx1* null mutants develop to the late embryonic stage due to maternal deposition of Syx1 mRNA. Development is arrested once maternal mRNAs are depleted and null embryos are paralyzed due to total absence of evoked and spontaneous SV release (Schulze et al., 1995). Structure-function studies targeting distinct regions of Syx1 and TS paralytic *syx1* mutants identified in forward genetic screens are consistent with an essential role for Syx1 in SV fusion (Littleton et al., 1998; Wu et al., 1999; Stewart et al., 2000; Fergestad et al., 2001; Lagow et al., 2007). However, *syx1* mutations in distinct regions of the protein differentially alter the amount of spontaneous versus evoked release, indicating Syx1 function can be altered to change either evoked or spontaneous SV fusion pathways. Consistent with an essential role for Syx1 in invertebrate SV fusion, null mutants in *C. elegans* Syx1 (*unc-64*) are immobile and lack detectable SV release (Ogawa et al., 1998; Saifee et al., 1998).

Genetic studies of the Drosophila Syb2 and SNAP-25 homologs have revealed phenotypes that are more challenging to interpret due to potential redundancy with other SNARE isoforms. Unlike Syx1, Syb2 function in SV and post-Golgi fusion is segregated between two Drosophila v-SNAREs, with c-Syb mediating post-Golgi fusion and n-Syb controlling SV release (Chin et al., 1993; DiAntonio et al., 1993; Broadie et al., 1995; Deitcher et al., 1998; Yoshihara et al., 1999). Although *n-Syb* null mutants show severe impairments in evoked release, a low rate of spontaneous fusion is preserved that indicates SV fusion is not eliminated. Consistently, high frequency stimulation elicits a low level of delayed evoked release (Yoshihara et al., 1999) and cleavage of n-Syb by tetanus toxin does not eliminate spontaneous fusion (Sweeney et al., 1995). *n-Syb* phenotypes can be rescued by overexpressing c-Syb, suggesting both proteins are capable of supporting SV release (Bhattacharya et al., 2002). Given overexpressed c-Syb supports relatively normal SV fusion in the absence of n-Syb, it is unclear why *n-Syb* mutants show defects primarily in evoked release. Perhaps endogenous neuronal c-Syb expression is too low to support evoked fusion, but high enough to contribute to residual spontaneous release. Alternatively, n-Syb may be specialized for evoked SV release, with spontaneous fusion supported by c-Syb and other Drosophila v-SNAREs (Littleton, 2000). Although no other v-SNARE beyond c-Syb has been shown to function in SV fusion in Drosophila, multiple v-SNAREs support spontaneous and asynchronous SV release at mammalian synapses (Ramirez et al., 2012; Lin et al., 2020). Similar to Drosophila, *C. elegans* null mutations in the Syb2 homolog (*snb-1*) are embryonic lethal, but retain uncoordinated movements that indicate a low level of residual SV release (Nonet et al., 1998).

Mutations in Drosophila *SNAP-25* indicate redundancy may also compensate for loss of t-SNARE function. The first mutant in Drosophila SNAP-25 was isolated as a TS paralytic allele caused by an amino acid substitution at a highly conserved residue (G50E) in the second SNARE motif of the protein (Rao et al., 2001). Upon exposure to the non-permissive temperature of 37°C, adult animals rapidly paralyze. *SNAP-25^*TS*^* mutant larvae show elevated evoked and spontaneous release at room temperature and impaired release at 37°C. A Syx1 TS mutant (*syx1^3–69^*; caused by a T254I substitution in the SNARE helix) displays a similar phenotype, indicating multiple t-SNARE mutations can alter SNARE dynamics in a manner that enhances fusion at lower temperatures and blocks release at elevated temperature (Littleton et al., 1998; Lagow et al., 2007; Bykhovskaia et al., 2013). While the mechanism underlying *SNAP-25*^TS^ release enhancement is unknown, the T254I mutation in Syx1 has been suggested to enhance release by altering interactions between the fusion clamp Cpx and the SNARE complex (Bykhovskaia et al., 2013), as well as promoting C-terminal domain SNARE zippering (Ma et al., 2015). Subsequent studies on SNAP-25 revealed null mutants cause pupal lethality, but do not affect SV release in larvae due to compensation from the t-SNARE homolog SNAP-24 (Vilinsky et al., 2002). Together, these data suggest SNAP-25 normally excludes endogenous SNAP-24 from participating in the SV SNARE complex, though SNAP-24 can support normal SV release when SNAP-25 is absent. *C. elegans* SNAP-25 null mutants (*ric-4*) have not been characterized electrophysiologically though they display locomotor defects that suggest RIC-4 is essential for normal synaptic function (Miller et al., 1996). In summary, genetic approaches in Drosophila and *C. elegans* indicate an essential role for Syx1 in all forms of SV fusion, with spontaneous release persisting in the absence of Syb2 and SNAP-25 likely due to compensation from non-SV SNAREs.

### Unc18 and Unc13 Restrict the Localization of SV SNARE Assembly to AZs by Regulating Syx1 Conformational Transitions

**FIGURE 2:**
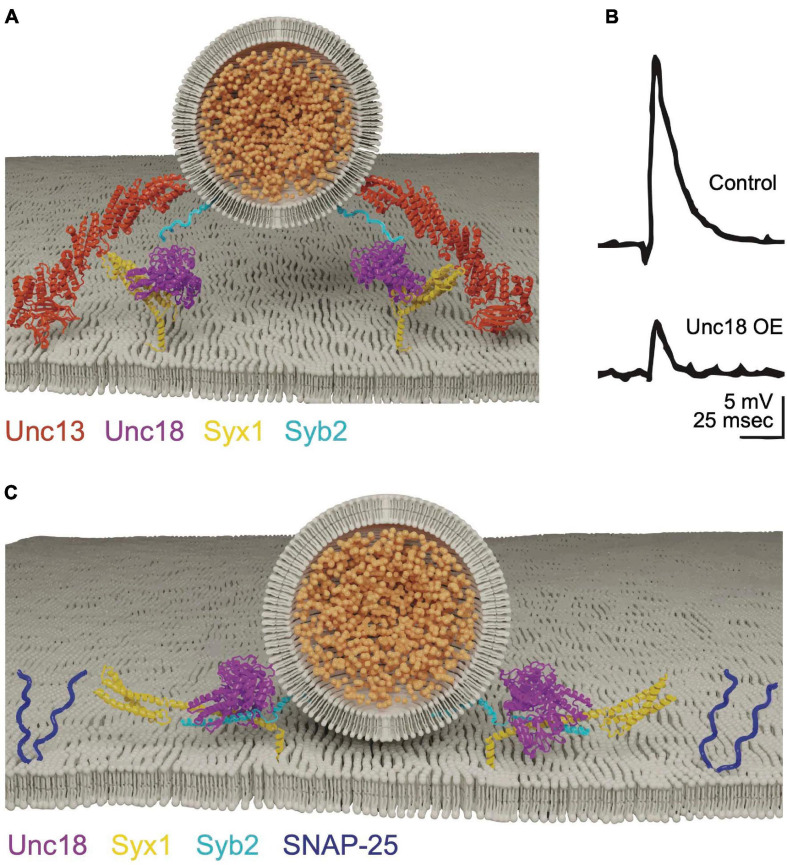
Unc13 and Unc18 chaperone SNARE complex assembly by regulating Syx1. **(A)** Unc18 holds Syx1 in a closed conformation prior to SNARE complex assembly. Unc13 bridges the SV and plasma membranes and interacts with Syx1 to drive transition to the open-Syx1 state. Adapted from Burkhardt et al. (2008); Liu et al. (2016); Xu et al. (2018). **(B)** Electrophysiological recordings of evoked responses at the Drosophila larval neuromuscular junction reveal overexpression of Unc18 impairs SV fusion. Adapted from Schulze et al. (1994). **(C)** Unc18 chaperones the assembly of Syb2 and Syx1 prior to SNAP-25 inclusion into the SNARE complex. Adapted from Burkhardt et al. (2008).

Although SNARE proteins are sufficient for membrane fusion *in vitro*, SRPs are required to regulate SNARE activity *in vivo*. Given SNARE complex formation is energetically favorable, the assembly process must be tightly controlled so it occurs at the right time and place for productive fusion (Rizo, 2018). The SM proteins (Sec1/Munc18, hereafter referred to as Unc18) and the AZ-localized Unc13 family are SRPs that control the subcellular localization of SNARE assembly. Unc18 is universally required for eukaryotic membrane fusion (Südhof and Rothman, 2009), while Unc13 functions only in regulated secretion (Aravamudan et al., 1999; Richmond et al., 1999). Both proteins act by controlling Syx1 availability and chaperoning SNARE complex assembly (Zhang and Hughson, 2021). Syx1 contains four α-helical domains with only the most C-terminal helix (termed the H3 domain) participating in SNARE complex formation (Fernandez et al., 1998; Sutton et al., 1998; Wu et al., 1999). The remaining N-terminal helices form a three-helix bundle called the H_abc_ domain that folds back onto the H3 SNARE motif to generate a monomeric four stranded coiled-coil bundle. The H_abc_ domain is separated from the H3 segment by a flexible hinge, allowing Syx1 to adopt an open or closed confirmation (Fernandez et al., 1998; Dulubova et al., 1999). In the closed state, the SNARE motif is locked into a grove along the length of the H_abc_ domain and blocked from participating in SNARE complex formation. When converted to the open state, the H3 domain is relieved of H_abc_ inhibition and SNARE complex formation can proceed. Point mutations in the hinge separating the H_abc_ and H3 domains bias Syx1 toward the open conformation (open-Syx1) and enhance SV fusion in *C. elegans* (Richmond et al., 2001; Gerber et al., 2008). These observations indicate the Syx1 closed conformation is an autoinhibitory feature that must be overcome for SV fusion to proceed, with Unc13 and Unc18 controlling this conformational switch (Figure 2A).

Unc18 proteins are cytosolic and bind to Syx1 in multiple conformational states (Hata et al., 1993; Pevsner et al., 1994; Yang et al., 2000; Dulubova et al., 2007; Khvotchev et al., 2007; Baker et al., 2015). These distinct binding interaction modes suggest Unc18 performs multiple roles in SNARE dynamics. Indeed, *in vivo* evidence indicates Unc18 both positively and negatively regulates SV release. The Drosophila Unc18 homolog ROP (Ras opposite) is essential for SV fusion, yet strongly inhibits both evoked and spontaneous release when overexpressed (Figure 2B; Harrison et al., 1994; Schulze et al., 1994; Wu et al., 1998). Like Syx1, ROP functions in all modes of cellular secretion and is required for SV and post-Golgi vesicle fusion (Harrison et al., 1994; DeBruhl et al., 2016). Unc18 proteins suppress Syx1 activity in part by holding the t-SNARE in its closed state (Pevsner et al., 1994; Yang et al., 2000). This interaction is required for transport of Syx1 through the secretory pathway, reducing its ability to form ectopic SNARE complexes at inappropriate times or subcellular locations (Rowe et al., 1999, 2001; Medine et al., 2007; McEwen and Kaplan, 2008). Overexpression of Unc18 is predicted to suppress neurotransmitter release by preventing formation of fusogenic SNARE complexes due to excessive inhibition of Syx1. Heterozygotes of *Unc18* null mutants also display reduced evoked and spontaneous fusion, indicating SV release is impaired under conditions where Unc18 levels are limiting (Wu et al., 1998). Together, these data indicate SV release is bi-directionally sensitive to Unc18 abundance, suggesting synaptic levels of the protein are finely tuned for optimal presynaptic output.

Unc18 must also play a positive role in release given *Unc18* null mutants show severe secretion defects (Harrison et al., 1994; Verhage et al., 2000; Weimer et al., 2003). Multiple positive effects of Unc18 on SV release have been described, including its ability to protect SNARE complexes from disassembly by NSF and α-SNAP. Assembly of the SNARE complex *in vitro* is blocked when NSF and α-SNAP are added, suggesting SNAREs must be protected from ongoing disassembly (Ma et al., 2013; Prinslow et al., 2019; Stepien et al., 2019). Addition of Unc18 and Unc13 to these *in vitro* assays restores the ability of SNAREs to trigger fusion, indicating the two proteins act in concert to ensure fusogenic SNARE zippering is not disrupted by premature disassembly. Unc18 also chaperones SNARE assembly by properly aligning individual SNARE helices during zippering of the 4-stranded helical bundle (Ma et al., 2013; Jiao et al., 2018). The SNARE complex is a coiled-coil structure divided into layers of hydrophobicity defined relative to the most central zero layer (Fasshauer et al., 1998). Misalignment of zippering decreases free energy released during SNARE assembly and alters the distance between fusing membranes (Fasshauer et al., 1998; Pobbati et al., 2006). *In vitro* data suggest Unc18 binds Syx1 and Syb2 in a prefusion intermediate where the two SNAREs are arrested in a partially zippered state and held in proper alignment prior to SNAP-25 arrival (Figure 2C; Jiao et al., 2018; Shu et al., 2020). This role of Unc18 in SNARE assembly is supported by crystal structures of several yeast homologs that hold individual v- and t-SNAREs in proper register (Baker et al., 2015). In summary, Unc18 likely supports SV fusion by templating SNARE complex assembly and inhibiting SNARE disassembly prior to fusion. How Unc18 transitions from inhibiting Syx1 availability by holding the protein in a closed conformation to templating Syx1 and Syb2 assembly is unclear, though Unc13 is hypothesized to regulate this transition *in vivo*.

Unc13 is one of several multidomain scaffold proteins enriched at presynaptic AZs. Unlike most AZ scaffolds, Unc13 is absolutely essential for both spontaneous and evoked release (Aravamudan et al., 1999; Augustin et al., 1999; Richmond et al., 1999). Unc13 contains C2, MUN and calmodulin binding domains that each have highly conserved binding interactions across evolution (Brose et al., 2000; Böhme et al., 2016). The lipid-binding C2 domains encoded at both termini of Unc13 enable simultaneous interaction with the SV and plasma membrane to facilitate SV capture (Liu et al., 2016). The MUN domain forms a long helical rod that extends from the AZ into the cytosol, similar to other vesicle tethering factors. The MUN domain plays a critical role in SV priming by converting Syx1 from its closed to open state, leading to subsequent v-SNARE engagement and SV docking (Betz et al., 1997; Li et al., 2011; Ma et al., 2011; Wang et al., 2011). Consistent with this model, open-Syx1 mutants rescue release defects in *C. elegans Unc13* nulls, indicating *Unc13* animals lack SV fusion due to insufficient conversion of Syx1 from its closed to open state (Richmond et al., 2001).

### Syt1 and Cpx Regulate SNARE Assembly to Control the Timing of Ca^2+^-Dependent Fusion

**FIGURE 3:**
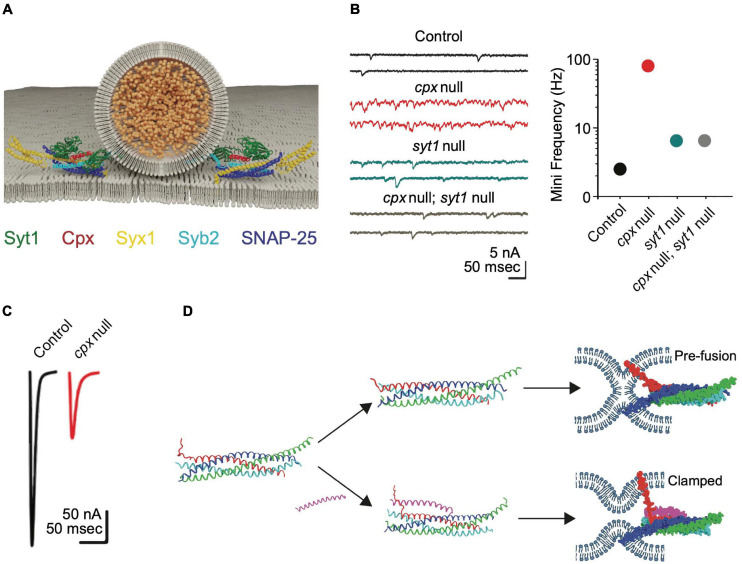
Cpx clamps the SNARE complex in a partially assembled state. **(A)** Cpx and Syt1 engage the SNARE complex on a shared binding interface to form a tripartite complex. Adapted from Zhou et al. (2017). **(B)** Electrophysiological recordings at Drosophila larval NMJs reveal *cpx* null mutants have a dramatically increased rate of spontaneous release. Spontaneous release is restored to near normal levels in *syt1; cpx* double mutants, indicating Syt1 is required for the elevated mini rate in *cpx* single mutants. Quantification of spontaneous release rate (mini frequency) is shown on the right for the indicated genotypes. **(C)** Evoked response amplitude is reduced in *cpx* null mutants, indicating Cpx is also required for efficient Ca^2+^-activated release. Panels **(B,C)** adapted from Jorquera et al. (2012). **(D)** Molecular-dynamics modeling suggests Cpx may clamp SNAREs in a partially assembled state by altering the confirmation of the C-terminus of Syb2 to prevent full SNARE zippering. Adapted from Bykhovskaia et al. (2013).

Membrane fusion during constitutive secretion occurs spontaneously, with SNARE complex zippering hypothesized to occur in a single step. In contrast, SNARE assembly during regulated secretion is predicted to arrest in a partially zippered state, allowing membrane fusion to be tightly coupled to Ca^2+^ influx. Progressive step-wise zippering of the SV SNARE complex is supported by studies of the clostridial neurotoxins tetanus and botulinum that cleave individual SNAREs (Breidenbach and Brunger, 2005). After SV docking and priming, only a subset of toxin serotypes can access SNAREs for cleavage at each conformational state generated by progressive zippering (Hayashi et al., 1994; Bajohrs et al., 2004). Intermediate energy states along the trajectory of SNARE zippering are also observed *in vitro* using optical tweezers, further suggesting SNAREs assemble and disassemble in a step-wise manner (Gao et al., 2012; Zorman et al., 2014). The synaptic SRPs Cpx and Syt1 provide a neuronal-specific mechanism to further stall SNARE zippering until elevated Ca^2+^ triggers full SNARE assembly (Figure 3A). Cpx acts during *trans-*SNARE complex formation to arrest assembly in the partially zippered state (Giraudo et al., 2006; Malsam et al., 2012; Bykhovskaia et al., 2013), with Syt1 triggering full zippering and synchronous evoked fusion in response to Ca^2+^ (Chapman, 2008; Südhof, 2013; Quiñones-Frías and Littleton, 2021). The mechanisms by which these proteins regulate SNARE assembly and fusion are still being defined, but several models link their biochemical activities with defects in release observed in mutants disrupting their function.

Cpx is small cytosolic α-helical protein identified through its binding affinity for the SNARE complex (McMahon et al., 1995). The protein is composed of an N-terminal accessory helix, a central SNARE-binding helix and an unstructured C-terminus that assembles into an amphipathic helix when bound to SV membranes (Pabst et al., 2000; Bowen et al., 2005; Xue et al., 2007; Cho et al., 2010; Kaeser-Woo et al., 2012; Buhl et al., 2013; Snead et al., 2014). Mouse *Cpx* mutants have decreased evoked release, suggesting the protein facilitates SV fusion (Reim et al., 2001). Subsequent *in vitro* assays indicated Cpx primarily functions to inhibit SNARE assembly and fusion (Giraudo et al., 2006; Malsam et al., 2020). In contrast to four *Cpx* genes in mammals, Drosophila contain a single *Cpx* that facilitates genetic analysis. Null mutants in Drosophila *Cpx* revealed both positive and negative functions in SV release, including a ∼100-fold increase in spontaneous fusion (Figure 3B) and a ∼50% decrease in evoked release (Figure 3C; Huntwork and Littleton, 2007). *C. elegans Cpx* null mutants display similar defects, indicating enhanced spontaneous fusion and decreased evoked release are conserved invertebrate phenotypes associated with loss of Cpx (Xue et al., 2009; Cho et al., 2010; Martin et al., 2011; Jorquera et al., 2012; Buhl et al., 2013; Iyer et al., 2013; Sabeva et al., 2017; Wragg et al., 2017). *Cpx* mutants also disrupt the speed of evoked release, with less synchronous fusion and increased release through the slower asynchronous pathway (Jorquera et al., 2012). In addition, Cpx participates in tethering SVs to release sites by interacting with the core AZ scaffolding protein Bruchpilot (BRP) (Scholz et al., 2019). Together, these observations indicate Cpx helps target SVs to release sites, facilitates the amount and speed of evoked release, and clamps SVs in a partially zippered state that limits spontaneous fusion.

In contrast to the dramatic increase in spontaneous fusion in invertebrate *Cpx* mutants, mouse *Cpx* knockouts do not display elevated spontaneous release (Xue et al., 2008, 2007; Yang et al., 2013; Chang et al., 2015; López-Murcia et al., 2019). However, mammalian Cpx is sufficient to clamp spontaneous release in both *C. elegans* and Drosophila *Cpx* mutants (Cho et al., 2010; Wragg et al., 2017), suggesting clamping properties are intrinsic to Cpx across phyla. Cpx3 is the most effective mammalian isoform for clamping SV fusion in Drosophila and *C. elegans Cpx* mutants. The primary difference between Cpx3 and other mammalian isoforms occurs in the C-terminus, suggesting this region harbors critical determinants for clamping fusion. Although it is unclear why mammalian synapses are more resistant to enhanced spontaneous release in *Cpx* mutants, Cpx can clamp SV fusion during the asynchronous phase of evoked release in mammals (Yang et al., 2010; Chang et al., 2015). This slower component of release occurs when Ca^2+^ levels are falling from their peak concentration that drives synchronous SV fusion. Therefore, higher baseline Ca^2+^ levels in invertebrate presynaptic terminals could account for the differences in Cpx clamping. Consistent with this hypothesis, presynaptic [Ca^2+^] can be reduced by long-term exposure to BAPTA and causes a ∼50% decrease in spontaneous release in Drosophila *Cpx* mutants (Jorquera et al., 2012). These data suggest Cpx clamping acts optimally at a slightly higher baseline [Ca^2+^], implying it may act in part by regulating the Ca^2+^ sensitivity of SV release.

Current data indicate the activating and inhibitory functions of Cpx can be genetically separated, though both require SNARE complex binding (Xue et al., 2007, 2010; Cho et al., 2010, 2014; Krishnakumar et al., 2011; Iyer et al., 2013). Several models for the inhibitory function of Cpx have been proposed. A “zig-zag” model based on structural evidence suggests the central helix of Cpx tucks into a partially zippered SNARE complex, while the accessory helix projects out at a 45-degree angle to bind a neighboring partial SNARE assembly (Kümmel et al., 2011). This mode would allow Cpx to bridge partial SNARE assemblies in an alternating zigzag chain sandwiched between docked SVs and the plasma membrane to clamp release before Ca^2+^ influx. Mutations predicted to abolish the zig-zag array have only mild effects on SV release in Drosophila, suggesting this binding mode is unlikely to represent the primary clamping configuration of Cpx (Cho et al., 2014). A second model from biochemical studies and molecular modeling suggests competition between Syb2 and Cpx for t-SNARE binding mediates clamping, with the Cpx N-terminal accessory helix binding partially assembled SNARE complexes in a grove between Syx1 and SNAP-25 in place of Syb2 (Figure 3D). This would allow Cpx to destabilize the final step of SNARE zippering by excluding Syb2 from the C-terminus of the SNARE complex (Bykhovskaia et al., 2013; Vasin et al., 2016; Brady et al., 2021). Although attractive, genetic analysis of *Cpx* and *n-Syb* mutations predicted to disrupt this mode of binding only partially disrupt clamping (Vasin et al., 2016). A modified version of the competition model has also been described where Syb2, a single helix of SNAP-25, and the Cpx accessory helix form a C-terminal helical bundle that displaces Syx1 from the SNARE complex at its C-terminus (Malsam et al., 2020). Mutations disrupting this binding mode do not affect evoked release but decrease the clamping efficiency for spontaneous fusion.

**FIGURE 4:**
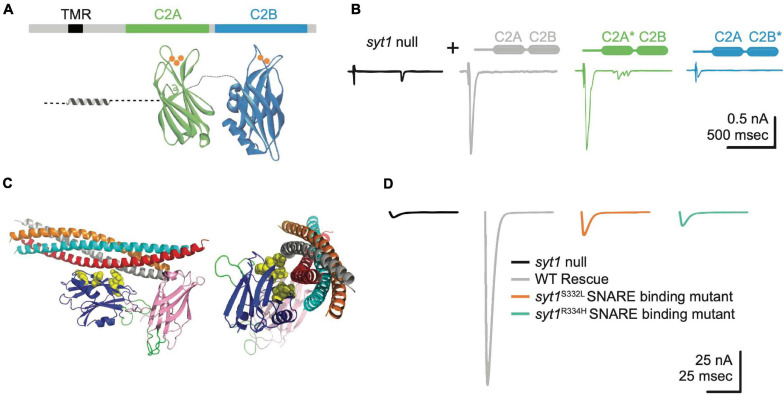
Syt1 binds Ca^2+^ and SNARE complexes to trigger synchronous SV fusion. **(A)** Syt1 is tethered to SV membranes via a transmembrane region (TMR) and has two Ca^2+^-binding C2 domains projecting into the cytosol. Ca^2+^ (orange) binds to polybasic loops projecting from each C2 domain, with C2A accommodating 3 Ca^2+^ ions and C2B binding 2 Ca^2+^ ions. **(B)** Two-electrode voltage clamp recordings demonstrate *syt1* nulls have dramatically impaired evoked synchronous release at Drosophila larval NMJs. Unlike rescue with wildtype (WT) *Syt1* (gray trace), transgenes with impaired C2B Ca^2+^ binding (C2B*, blue) severely impair evoked release while C2A Ca^2+^ binding mutants (C2A*, green) fail to prevent the enhanced asynchronous release observed in nulls (black). Adapted from Yoshihara et al. (2010). **(C)** The Syt1 C2B domain (blue) also interacts with the SNARE complex at a primary interface independent of the Cpx-associated tripartite binding site. Critical residues coordinating binding of the Syt1 C2B domain to the SNARE complex are highlighted in yellow and were identified from crystal structures of the complex and genetic screens in Drosophila. **(D)** Disrupting Syt1-SNARE binding at the primary interface with two independent alleles mimics the *syt1* null phenotype. Panels **(C,D)** adapted from Guan et al. (2017).

Regardless of its clamping configuration, enhanced spontaneous release in *Cpx* mutants is abolished in Drosophila *Cpx, Syt1* double mutants (Figure 3B; Jorquera et al., 2012). Syt1 is a SV protein with tandem C2 domains (C2A and C2B, Figure 4A) that bind ∼ five Ca^2+^ ions via negatively charged aspartate residues encoded within protruding C2 loops (Ubach et al., 1998; Chapman, 2008). Ca^2+^ binding neutralizes the negative charge of these loops to allow C2•Ca^2+^ to partially insert into the plasma membrane (Davletov and Südhof, 1993; Chapman and Davis, 1998; Fernandez et al., 2001; Ubach et al., 2001). Ca^2+^ binding to the C2B domain of Drosophila Syt1 is critical for promoting evoked release (Figure 4B), with C2A-Ca^2+^ playing a supporting role (Littleton et al., 2001a; Mackler et al., 2002; Paddock et al., 2008, 2011; Yoshihara et al., 2010; Striegel et al., 2012; Lee et al., 2013; Bowers and Reist, 2020). The genetic interactions between *Syt1* and *Cpx* suggest loss of Cpx may disrupt Syt1’s ability to link its fusion activation to Ca^2+^ binding. Following loss of Cpx, Syt1 may constitutively activate SNARE-dependent fusion in a Ca^2+^-independent manner, leading to elevated spontaneous fusion rates.

Regulation of Syt1 activity is also likely to contribute to Cpx’s positive role in promoting fusion as mutations in either gene cause similar SV release defects, though *Cpx* phenotypes are generally milder than those found in *Syt1* (Jorquera et al., 2012). Both *Cpx* and *Syt1* mutants show impaired evoked synchronous release while enhancing the number of SVs released through the slower asynchronous pathway (Mackler et al., 2002; Yoshihara and Littleton, 2002; Saraswati et al., 2007; Yoshihara et al., 2010; Paddock et al., 2011; Jorquera et al., 2012; Striegel et al., 2012; Lee and Littleton, 2015; Guan et al., 2020; Shields et al., 2020). Both mutants show an increased rate of spontaneous fusion (Littleton et al., 1993, 1994; DiAntonio and Schwarz, 1994; Huntwork and Littleton, 2007; Lee et al., 2013), with Cpx playing the primary role in clamping release at invertebrate terminals and Syt1 assuming this function at mammalian synapses. The Ca^2+^ sensitivity of evoked release is also reduced in either mutant (Littleton et al., 1994; Jorquera et al., 2012), suggesting a greater number of Ca^2+^ ions are required to fuse SVs in their absence. Evoked release defects in *Cpx* mutants can be partially rescued with elevated extracellular [Ca^2+^], arguing Ca^2+^ sensitivity is impaired but not abolished (Jorquera et al., 2012). In contrast, *Syt1* mutants display severely impaired release across the entire [Ca^2+^] range (Littleton et al., 1994; Yoshihara and Littleton, 2002). Finally, both mutants reduce the size of the readily releasable SV pool and alter the speed of SV fusion in a Ca^2+^-dependent manner (Yoshihara and Littleton, 2002; Mace et al., 2009; Jorquera et al., 2012; Lee and Littleton, 2015). Recent structural evidence provides a potential model explaining why these SRPs phenocopy each other. Syt1 and Cpx bind the outer surface of the SNARE complex to form a split but continuous α-helix as part of a “tripartite complex” (Figure 3A), suggesting Syt1 and Cpx may form a single regulatory unit that reduces the energy barrier needed for full SNARE zippering (Trimbuch and Rosenmund, 2016; Zhou et al., 2017).

Beyond the tripartite SNARE binding site with Cpx, a primary SNARE complex binding interface on a distinct surface of the Syt1 C2B domain is critical for triggering SV release (Zhou et al., 2015b; Guan et al., 2017). The five key residues that form the primary binding site based on structural data were independently identified in a genetic screen for *Syt1* mutants in Drosophila (Figure 4C), indicating this interface is highly conserved and essential for Syt1 function (Guan et al., 2017). Disrupting SNARE binding at this site phenocopies *Syt1* null mutants in critical ways (Figure 4D), including loss of synchronous fusion and elevated rates of asynchronous and spontaneous release. Current models for Syt1’s role in fusion suggest SNARE binding, together with Ca^2+^-independent lipid interactions mediated through a polybasic stretch on a separate C2B surface, sandwich Syt1 between the plasma membrane and the partially assembled SNARE complex. This positions the Ca^2+^ binding loops of Syt1’s C2 domains close to the plasma membrane, enabling rapid membrane insertion of the loops following Ca^2+^ entry to trigger a conformational rotation that pulls the two fusing membranes together and initiates full SNARE zippering (Quiñones-Frías and Littleton, 2021). Ca^2+^-dependent conformational changes in Syt1 may also dislodge Cpx to facilitate conversion from the *trans-* to *cis*-SNARE complex to drive the final fusion reaction. Displacement of Cpx would provide binding sites for α-SNAP to initiate subsequent NSF-mediated SNARE complex disassembly. Together, these data indicate Syt1 and Cpx cooperate to prevent full SNARE zippering during SV priming and later activate full fusion following Ca^2+^ influx.

### The AAA+ ATPase NSF Disassembles SNARE Complexes to Support Continual SV Cycling

**FIGURE 5:**
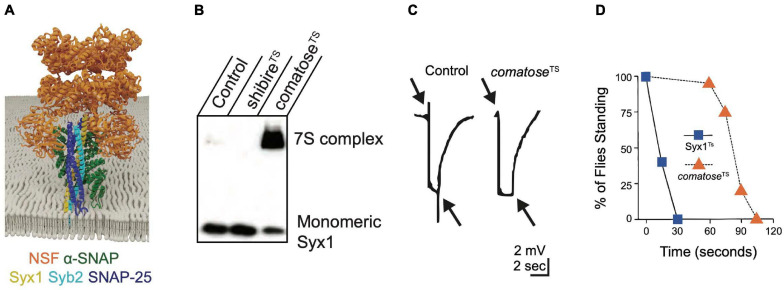
NSF and α-SNAP disassemble the *cis*-SNARE complex to maintain a pool of available free SNAREs for sustained release. **(A)** α-SNAP binds assembled SNARE complexes, enabling NSF to form a hexamer around the SNARE complex to initiate disassembly. Adapted from Zhao et al. (2015). **(B)** Western blot of Drosophila brain extracts with anti-Syx1 antisera demonstrates 7S complexes accumulate at the restrictive temperature in *comatose*^TS^ mutants at the expense of monomeric Syx1. Heat-shocked *shibire*^TS^ mutants have reduced 7S complex compared to controls, indicating NSF continues to disassemble SNARE complexes within the plasma membrane when SVs are depleted. **(C)** Electroretinograms recorded from Drosophila TS *comatose* mutants reveal a loss of on and off transients (arrows) at elevated temperatures that occurs secondary to disrupted synaptic transmission from photoreceptors. Panels **(B,C)** adapted from Littleton et al. (2001b). **(D)** Drosophila TS mutants in *Syx1* show rapid behavioral paralysis compared to the slower time-course for *comatose* TS mutations in NSF. Adapted from Littleton et al. (1998).

NSF proteins are highly conserved AAA+ ATPases that disassemble SNARE complexes for both constitutive and regulated membrane trafficking (Wilson et al., 1989; Pallanck et al., 1995a). Disassembly of the SNARE complex supplies the energy input required for membrane fusion by returning SNAREs to their disordered state for future rounds of assembly (Block et al., 1988; Wilson et al., 1992; Littleton et al., 1998). The *cis*-SNARE complex (also called the 7S complex based on its gradient sedimentation) is highly stable and resistant to SDS denaturation, indicating a large input of cellular energy is required to break the complex apart (Fasshauer et al., 2002). Full dissociation of the SNARE complex is estimated to consume between 12 and 50 ATP molecules, generating 65 *k*B*T* of free energy that can be used to drive membrane fusion during future cycles of SNARE assembly (Cipriano et al., 2013; Yoon and Munson, 2018). NSF proteins are composed of an N-terminal domain and two AAA+ ATPase domains termed D1 and D2 (Tagaya et al., 1993; White et al., 2018). The D2 domains promote multimerization of NSF into hexamers that assemble around a single SNARE complex via interactions between the NSF N-terminal domains and SNAP proteins that preferentially associate with assembled SNARE complexes (Zhao et al., 2015; White et al., 2018). Once the SNAP/NSF/SNARE complex is formed (termed the 20S complex, Figure 5A), the D1 domains of NSF use ATP hydrolysis to twist the four-helical SNARE bundle opposite to its assembled orientation until individual SNAREs are sufficiently destabilized to disassociate (Cipriano et al., 2013; Ryu et al., 2015; Zhao et al., 2015). Whether SNARE disassembly by NSF occurs in a multi-step process or all at once remains contentious. One model based on the processive mechanism of the bacterial AAA+ ATPase ClpXP suggests NSF may drive SNARE disassembly by progressing along the assembled SNARE complex in discrete steps that each require ATP hydrolysis (Saunders et al., 2020). An alternate model supported by *in vitro* single molecule assays suggests NSF may use a spring-loaded trigger mechanism to disassemble the SNARE complex in a single round of ATP hydrolysis (Ryu et al., 2015).

The Drosophila genome encodes two NSF proteins. NSF1 (*comatose*) mediates neuronal SNARE complex disassembly (Ordway et al., 1994; Boulianne and Trimble, 1995; Pallanck et al., 1995b) and NSF2 functions more broadly, including in the post-synaptic compartment (Golby et al., 2001; Stewart et al., 2002). TS behavioral screens in Drosophila uncovered numerous mutants in NSF1 that were originally named after their strong paralytic phenotype (*comatose*) (Siddiqi and Benzer, 1976; Pallanck et al., 1995a). Many *comatose* TS alleles result from single amino acid changes within a hinge region of the D1 domain that may impair the ability of NSF to twist the SNARE complex apart (Littleton et al., 2001b). Although restrictive temperatures are predicted to immediately disrupt NSF function, adult *comatose* animals behave normally for ∼ one minute before paralysis (Figure 4B). Accumulation of assembled 7S SNARE complexes (Figure 5B) and a progressive impairment of synaptic transmission within the visual system (Figure 5C) mirror the time course for paralysis (Littleton et al., 1998, 2001b; Tolar and Pallanck, 1998; Sanyal et al., 1999, 2001). These data suggest depletion of free SNAREs available to engage in SNARE complex assembly occurs after several rounds of SV fusion following loss of NSF1 function, leading to disrupted synaptic transmission and subsequent behavioral paralysis. The time course for recovery of *comatose* mutants correlates with the duration of the prior heat shock, likely due to the slow kinetics of NSF-mediated SNARE disassembly and the increasing depletion of free SNAREs as animals are maintained for longer periods at restrictive temperatures. The delayed onset and slow recovery from behavioral paralysis in NSF TS mutants contrasts with the rapid onset and recovery observed in Syx1 TS mutants (*Syx1^3–69^*, Figure 5D). These kinetic differences highlight the requirement of Syx1 for immediate SV fusion and the large pre-existing pools of free SNAREs and SVs available to support ongoing release for several minutes after NSF inactivation (Littleton et al., 1998).

Although *comatose* mutations cause adult paralysis and synaptic transmission defects, the role of NSF at larval NMJs has been difficult to ascertain. *NSF1* null mutants are lethal, but die over a developmental window that spans from late embryogenesis to the pharate adult stage (Golby et al., 2001; Littleton et al., 2001b; Sanyal and Krishnan, 2001). *NSF1* null larvae display no obvious transmission defects at the NMJ even though lethality is rescued when NSF1 is re-expressed in the nervous system. *NSF2* null mutants die during early larval development and are rescued by re-expression of NSF2 in mesodermal tissues, suggesting this isoform is predominantly active within muscles and other non-neuronal cells (Golby et al., 2001). NSF2 may also have SNARE-independent presynaptic functions given *NSF2* mutants have defects in SV mobility due to decreased presynaptic actin filament assembly (Nunes et al., 2006). Overexpression of NSF2 in the nervous system is sufficient to rescue release defects in *NSF1* mutants, suggesting differences in expression pattern and abundance are likely to account for their unique phenotypes. At the adult NMJ, *comatose* mutants display normal baseline synaptic transmission and a progressive activity-dependent reduction in evoked release during repetitive stimulation (Kawasaki et al., 1998; Kawasaki and Ordway, 1999). Together with an accumulation of docked SVs observed by EM at restrictive temperatures, these data suggest disassembly of SNARE complexes by NSF helps maintain SVs in a readily releasable state. Excess SNARE complexes in *comatose* mutants accumulate in both the presynaptic plasma membrane and on SVs (Tolar and Pallanck, 1998; Sanyal et al., 1999, 2001; Littleton et al., 2001b). Together, these data indicate NSF can break apart SNARE complexes in the plasma membrane that accumulate following fusion, as well as those already on SVs, to maintain a pool of free Syb2 to participate in *trans-*SNARE complex formation.

NSF relies on SNAP proteins to bind assembled SNARE complexes (Clary et al., 1990; Vivona et al., 2013). α-, β-, and γ-SNAP were initially purified from brain extracts based on their ability to promote NSF binding to SNARE-rich membranes. α-SNAP is the most extensively characterized and mediates association of NSF with the 7S SNARE complex *in vitro* and *in vivo* (Söllner et al., 1993; Barnard et al., 1997; Vivona et al., 2013; Zhao et al., 2015). The α-SNAP/SNARE complex binding interface includes residues from all SNAREs, suggesting it only recognizes assembled SNARE complexes (Whiteheart et al., 1992; Wilson et al., 1992; Marz et al., 2003). NSF hexamers bind SNAREs via four α-SNAP proteins that form a bridge between the SNARE complex and the NSF N-terminal domains (Zhao et al., 2015; Zhou et al., 2015a; Zhao and Brunger, 2016; White et al., 2018; Huang et al., 2019). *In vitro* evidence suggests NSF and α-SNAP indiscriminately disassemble SNARE complexes regardless of individual SNARE composition, suggesting they provide the energy for most cellular fusion reactions (Vivona et al., 2013; Zhao et al., 2015; Zhao and Brunger, 2016). Although the *in vitro* function of α-SNAP in SNARE disassembly has been well characterized, *in vivo* roles have not been extensively studied. In *C. elegans*, synaptic phenotypes resulting from NSF and α-SNAP mutations have not been reported, but Drosophila *α-SNAP* null mutants are embryonic lethal (Ordway et al., 1994; Babcock et al., 2004). Similar to Syx1 and Unc18, Drosophila *α-SNAP* mutants show defects in membrane trafficking in many cell types that indicate α-SNAP function is broadly required (Babcock et al., 2004). Like *comatose* mutants, *α-SNAP* hypomorphic alleles accumulate assembled 7S SNARE complexes but show no defects in synaptic transmission at larval NMJs (Babcock et al., 2004).

The functions of β- and γ-SNAP are less clear. α-SNAP is the only SNAP that restores *in vitro* fusion deficits in yeast *sec17* (α-SNAP) mutants, suggesting SNAPs are not fully redundant (Clary et al., 1990; Griff et al., 1992; Peter et al., 1998). β-SNAP is a vertebrate-specific paralog of α-SNAP generated by a gene duplication event occurring after divergence of vertebrates and invertebrates. γ-SNAP is dissimilar in sequence to both α- and β- SNAP and may play a supportive role in NSF/SNARE complex dynamics (Whiteheart et al., 1992; Peter et al., 1998; Stenbeck, 1998). Many invertebrates lack a γ-SNAP homolog, though Drosophila has two γ-SNAP genes that have not been characterized. Although no γ-SNAP mutant has been reported in any species, RNAi knockdown of γ-SNAP indicates the protein mediates disassembly of an endosomal SNARE complex, suggesting a canonical SNAP function (Inoue et al., 2015). Although the requirement of NSF and SNAPs for SNARE complex disassembly has been well defined, the full complement of *in vivo* functions mediated by these proteins remains poorly characterized.

### Multiple Modes of Endocytosis Mediate SV and SNARE Recycling

The SV and presynaptic plasma membranes become continuous during fusion, resulting in a temporary disruption in the spatial segregation of proteins. Many neurons can continue to release SVs for minutes to hours under high exocytotic demand, releasing far more SVs than observed in synaptic terminals by EM (Ceccarelli et al., 1973). To support further rounds of release, membrane proteins must be re-segregated and SV material selectively internalized to form new vesicles (Dittman and Ryan, 2009; Gan and Watanabe, 2018; Chanaday et al., 2019). SNARE disassembly by NSF is also required to free v-SNAREs from plasma membrane t-SNAREs after fusion. SNARE disassembly by NSF is hypothesized to occur in part at peri-active zones (PAZ), a presynaptic endocytotic domain surrounding AZs where SV material is retrieved from the plasma membrane (Estes et al., 1996; Littleton et al., 2001b; Rodal et al., 2008; Yu et al., 2011; Harris and Littleton, 2015; Maritzen and Haucke, 2018; Guan et al., 2020). Live imaging of NSF and α-SNAP show they redistribute from the cytoplasm to the peri-active zone (PAZ) to bind post-fusion SNARE complexes in Drosophila *comatose* mutants (Yu et al., 2011). Although endocytosis and SNARE disassembly can act within the same membrane compartment, how NSF activity is spatially and temporally coordinated with endocytosis is unknown. Three popular models have been proposed for SV endocytosis, including “kiss-and-run” endocytosis, ultrafast endocytosis and Clathrin-mediated endocytosis (CME) (Ceccarelli et al., 1973; Heuser and Reese, 1973; Fesce et al., 1994; Watanabe et al., 2013a,b).

Kiss-and-run is conceptually the simplest way to recover SV membrane proteins, with SNARE zippering causing brief fusion pore formation that releases neurotransmitters before the pore is quickly re-closed (Cremona and De Camilli, 1997; Rizzoli and Jahn, 2007; Chanaday et al., 2019). As such, SV material is never lost to the plasma membrane and SVs are immediately recovered without losing their identity. It is unclear how NSF-mediated disassembly works in this pathway given *cis*-SNARE complexes would not form in a fused membrane. Kiss-and-run has been documented for dense core vesicle (DCV) cargo release and for some SVs at a few central mammalian synapses (Artalejo et al., 1998; Aravanis et al., 2003; Xia et al., 2009; Zhang et al., 2009; Wen et al., 2017). However, experimental evidence does not support this mechanism at invertebrate synapses given kiss-and-run is a Clathrin-independent process (Henkel and Almers, 1996; Artalejo et al., 1998). Acute inactivation of Clathrin at Drosophila NMJs abolishes sustained release during repetitive stimulation and is accompanied by complete loss of SVs (Heerssen et al., 2008; Rodal and Littleton, 2008).

In contrast to kiss-and-run, CME enables SVs to be directly recovered from the plasma membrane after full collapse through progressive membrane invagination into reformed vesicles. Clathrin is a cytosolic protein that forms trimeric Y-shaped triskelions that progressively deform the plasma membrane by assembling on endocytic membrane patches to generate coated pits (Ungewickell and Branton, 1981; Smith et al., 1998). Further assembly shapes these pits into Clathrin-caged spheres that are budded from the plasma membrane (Heuser and Reese, 1973; Takei et al., 1996). GTP hydrolysis by the endocytic protein Dynamin provides energy to release nascent SVs from the plasma membrane by oligomerizing around the invaginating membrane stalk and pinching it to induce membrane fission (Hinshaw and Schmid, 1995; Sweitzer and Hinshaw, 1998; Ford et al., 2011). Hypomorphic alleles of the Drosophila Dynamin homolog *Shibire* were isolated in TS paralytic screens (Poodry et al., 1973; Koenig and Ikeda, 1989; Chen et al., 1991; van der Bliek and Meyerowitz, 1991; van de Goor et al., 1995; Delgado et al., 2000). *Shibire* mutants show fast synaptic depression and SV depletion at elevated temperatures, suggesting Dynamin is required to recover SVs for sustained release (Koenig and Ikeda, 1989; Delgado et al., 2000; Kawasaki et al., 2000; Wu et al., 2005).

Clathrin assembly is triggered by cytosolic adaptor proteins that recognize and cluster SV material into endocytic membrane patches (Haucke et al., 2011). Each SV protein is presumed to directly or indirectly associate with the general endocytic adaptor protein complex 2 (AP2) for retrieval from the membrane, with interactions between SV proteins likely contributing to AP2 recognition (Bennett et al., 1992a; Wittig et al., 2021). Syt1 is captured by the AP2 adaptor complex and Stonin2, while Syb2 is internalized by indirect AP2 association through the Clathrin adaptor AP180 (Zhang et al., 1994, 1998; Chapman et al., 1998; Nonet et al., 1999; Haucke et al., 2000; Littleton et al., 2001a; Martina et al., 2001; Walther et al., 2004; Bao et al., 2005; Diril et al., 2006; Kaempf et al., 2015). Mutations in the core α-adaptin subunit of the Drosophila AP2 complex cause embryonic lethality, with disrupted endocytosis and loss of SVs (González-Gaitán and Jäckle, 1997). Mutations in AP2 proteins in *C. elegans* also disrupt synaptic transmission and reduce SV numbers by up to 70%, with accumulation of large membrane vacuoles within synaptic terminals (Gu et al., 2008, 2013; Mullen et al., 2012). Loss of the Drosophila AP180 protein LAP causes a reduction in SV number, accumulation of cytosolic cisternae and increased SV size (Zhang et al., 1998), similar to mutants of the *C. elegans* AP180 homolog Unc11 that display an accumulation of Snb-1 (Syb2 homolog) on the plasma membrane, impaired neurotransmitter release and enlarged SVs (Nonet et al., 1999). NSF-mediated disassembly of the SNARE complex would expose Syb2 to AP180, providing one potential switch coupling SNARE disassembly to SV endocytosis. Neurotransmission is not fully eliminated in *AP180* mutants, suggesting Syb2 can be retrieved from the plasma membrane via another mechanism. The abundant SV protein Synaptophysin simultaneously binds Syb2 and AP2 to provide a secondary pathway for SV internalization (Pennuto et al., 2003; Yelamanchili et al., 2005; Bonanomi et al., 2007; Gordon and Cousin, 2014, 2016; Gordon et al., 2016, 2011). Syx1 cannot bind Syb2 while associated with Synaptophysin, suggesting Syb2 would only be available for endocytosis recruitment after SNARE complex disassembly (Edelmann et al., 1995; Siddiqui et al., 2007). In addition, Synaptophysin may act to chaperone Syb2 and prevent premature SNARE complex re-assembly. Given the lack of a Synaptophysin homolog in Drosophila (Stevens et al., 2012), additional mechanisms to support v-SNARE endocytosis and chaperoning likely exist.

Ultrafast endocytosis is a newly proposed mechanism for Dynamin-dependent SV formation where the plasma membrane immediately buckles into the cytoplasm when SVs fuse (Watanabe et al., 2013a,b; Gan and Watanabe, 2018). Optically stimulated *C. elegans* motoneurons fixed within 25 milliseconds of SV release show large membrane invaginations at the periphery of the AZ that quickly resolve into cytosolic endosomes (Watanabe et al., 2013a). Following Dynamin activity, Clathrin-mediated fission of these endosomes generates new SVs. However, it is currently unclear whether the rapidly invaginated membrane compartments observed by EM contain SV proteins that were just exocytosed. This form of endocytosis may primarily act to relieve plasma membrane tension by internalizing lipids rather than fused SV proteins. Given the slow kinetics of NSF-mediated SNARE disassembly and the rapid time-course of ultrafast endocytosis, it is unlikely NSF could disassemble *cis*-SNARE complexes prior to plasma membrane internalization via this mechanism. As such, ultrafast endocytosis may internalize SV material lost during prior rounds of fusion. Indeed, some SV cargo like Syt1 and Syb2 are localized to the plasma membrane in resting mammalian neurons (Fernández-Alfonso et al., 2006), potentially allowing ultrafast endocytosis to draw from a pool of SV material normally found on the plasma membrane.

### SNAREs, Rabs, and Rab Effectors Contribute to Target Specificity for Membrane Fusion

Following endocytosis, SVs navigate a host of non-target presynaptic membrane compartments as they traffic back to AZs. Dozens of t- and v-SNAREs are encoded in eukaryotic genomes, implying a large combinatorial assortment of possible SNARE complexes across different subcellular compartments. Only a subset of these theoretical complexes associate *in vitro* and *in vivo* to promote membrane fusion (Söllner et al., 1993; McNew et al., 2000a), suggesting cognate SNARE binding contributes to fusion specificity. SRPs also facilitate specificity in membrane trafficking, with Rab proteins and their effectors acting in vesicle-target recognition upstream of SNARE interactions. Rab proteins are monomeric GTPases that specify membrane identity by associating with one or a small subset of intracellular compartments via two cysteine-linked prenyl groups (Stenmark, 2009). These lipid anchors are exposed in the Rab GTP-bound active state and occluded following GTP hydrolysis (inactive GDP-bound state), allowing Rabs to cycle on and off membranes in a GTP-dependent manner (Pereira-Leal et al., 2001; Stenmark and Olkkonen, 2001). Rabs sculpt intracellular membrane composition by recruiting and activating effector proteins that include tethering factors, SNAREs, cytoskeleton modifiers, lipid kinases/phosphatases, endocytic proteins, and protein scaffolds (Grosshans et al., 2006; Stenmark, 2009). Among this group, tethering factors form critical Rab effectors for mediating membrane fusion specificity. These multimeric protein complexes project farther into the cytosol than SNARE proteins, identifying and luring vesicles via specific affinity for vesicular Rabs or SNAREs (Whyte and Munro, 2002; Yu and Hughson, 2010; Witkos and Lowe, 2015; Spang, 2016). Tethering complexes are phylogenetically diverse and include the Exocyst, Golgin, CORVET, and HOPS complexes, each tethering a distinct vesicle type to their cognate target membrane. After tethering factors bring vesicles to the appropriate target compartment, *trans-*SNARE assembly docks and primes them for fusion.

At the synapse, AZ components project into the cytoplasm to engage and recruit SVs to release sites via several large scaffolding proteins (Zhai and Bellen, 2004; Südhof, 2012; Ghelani and Sigrist, 2018). One of the most prominent AZ scaffold proteins in Drosophila is the ELKS/CAST-like protein BRP (Wagh et al., 2006). *Brp* null mutants lack the electron-dense T-bar structure at Drosophila AZs and have reduced evoked release secondary to decreased AZ Ca^2+^ channel density (Kittel et al., 2006; Wagh et al., 2006). *Brp* mutants lacking only the cytosolic C-terminus of the protein (*Brp*^*nude*^) have normal AZ dense body projections and Ca^2+^ channel clustering, but fail to accumulate SVs that normally surround the AZ due to defective Cpx binding (Hallermann et al., 2010; Scholz et al., 2019). *Brp*^*nude*^ mutants display normal evoked release at low frequency, but severely reduced release following repetitive stimulation. These data indicate BRP is not an essential tethering factor for SV docking and priming, but clusters SVs for fast refilling of release sites to support sustained activity.

Among the AZ proteins that mediate SV targeting, RIM tethers vesicles to release sites via its interaction with Rab3 (Wang et al., 1997; Betz et al., 2001; Koushika et al., 2001; Schoch et al., 2002; Han et al., 2011). *RIM* mutants have severely impaired evoked release accompanied by a dramatic reduction in SV docking (Gracheva et al., 2008; Han et al., 2011; Graf et al., 2012; Kushibiki et al., 2019; Oh et al., 2021). Together with the AZ protein SYD-2/Liprin, the *C. elegans* RIM homolog Unc10 forms filamentous projections that extend from the AZ to capture and tether Rab3 bound SVs at release sites (Gracheva et al., 2008; Stigloher et al., 2011). *Rab3* mutants also show defects in SV recruitment, but have more modest impairments in evoked release (Geppert et al., 1997; Nonet et al., 1997; Schlüter et al., 2006; Gracheva et al., 2008; Graf et al., 2009). Drosophila and *C. elegans RIM/Rab3* double mutants do not have enhanced release defects, suggesting they operate in a similar pathway. Beyond SV tethering, Drosophila *Rab3* mutants have defects in AZ maturation, with a subset of release sites lacking late AZ scaffold proteins required for efficient evoked release (Graf et al., 2009). This AZ maturation phenotype is not observed in *RIM* mutants, suggesting Rab3 interfaces with other Rab effector proteins to deliver AZ components during synaptic development.

RIM is hypothesized to partner with Unc13 to transition SVs from a tethered state to the downstream Unc18/Unc13-dependent priming mechanism (Liu et al., 2019). RIM and Unc13 display highly conserved interactions mediated by a C2 domain in Unc13 and a zinc finger domain of RIM. Disruption of the RIM/Unc13 interaction reduces the number of fusion competent SVs, suggesting efficient SV recruitment requires engagement with Unc13 (Betz et al., 2001). Homodimerization of Unc13 in *C. elegans* generates an autoinhibitory state that is relieved by RIM binding prior to SV priming (Liu et al., 2019). These biochemical interactions suggest a model where Unc13 autoinhibition is relieved by RIM before it primes Syx1 for SNARE complex assembly. Given the RIM zinc finger domain also interacts with Rab3 (Betz et al., 2001; Weimer et al., 2006; Liu et al., 2019), priming may be coupled to SV arrival through competition for RIM binding between Unc13 and SV-bound Rab3. Release of RIM from Rab3 would allow it to activate Unc13 for Syx1 priming, with open Syx1 engaging Unc18 on a distinct interaction surface that templates the onset of SNARE complex assembly between Syx1 and SV-localized Syb2. Release defects in both *Unc10/RIM* and *Unc13* can be rescued by the open-Syx1 mutation (Tien et al., 2020), consistent with RIM-mediated SV tethering facilitating downstream SNARE-dependent SV priming.

### Tomosyn Acts as a Decoy SNARE to Negatively Regulate SNARE Complex Assembly

**FIGURE 6:**
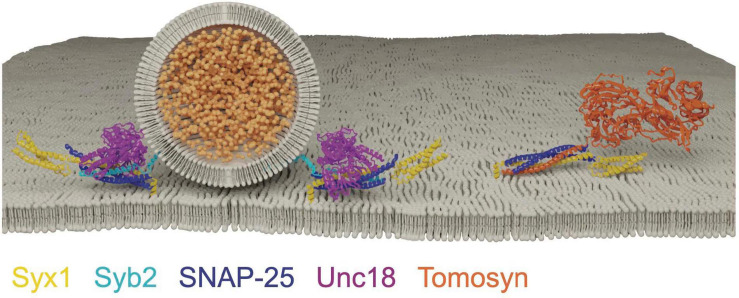
Tomosyn forms a decoy SNARE complex with Syx1 and SNAP-25 in an Unc18 and Syb2 independent manner. Adapted from Pobbati et al. (2004); Hattendorf et al. (2007).

SNARE proteins incompatible with membrane fusion, known as decoy SNAREs, provide an additional mechanism to ensure regulated SNARE assembly. Several decoy SNAREs have been described in mammals, but Tomosyn is the only known invertebrate decoy SNARE (Masuda et al., 1998; Lao et al., 2000; Scales et al., 2002; Gerst, 2003; Ashery et al., 2009). Tomosyn was originally identified for its ability to displace Syx1 from Unc18, and suggested to engage Syx1 and SNAP-25 in an intermediate stage of SNARE assembly preceding *trans-*SNARE complex formation (Fujita et al., 1998; Masuda et al., 1998; Pobbati et al., 2004). Subsequent experiments revealed Tomosyn prevents Syb2 binding to t-SNAREs, suggesting this complex is not a prefusion intermediate but may rather inhibit productive SNARE complex assembly (Yokoyama et al., 1999). Tomosyn is a large cytoplasmic protein with a Syb2-like R-SNARE motif at its C-terminus that forms a four-helical SNARE complex with synaptic t-SNAREs (Figure 6). However, the absence of a membrane anchor in Tomosyn prevents formation of fusogenic SNARE complexes. At the N-terminus, Tomosyn contains WD40 repeats organized into a propeller-like scaffold with homology to L(2)GL proteins (Lehman et al., 1999; Williams et al., 2011). *In vivo* studies suggest Tomosyn can also interact with SNAREs beyond its R-SNARE motif, as the yeast Tomosyn-like protein Sro7 lacks a SNARE motif and uses its scaffold domain to coordinate vesicle docking with SNARE assembly by binding the Sec9 t-SNARE (Lehman et al., 1999; McEwen et al., 2006; Yizhar et al., 2007; Yamamoto et al., 2010).

Tomosyn overexpression reduces both constitutive and regulated secretion in a diverse set of eukaryotes, suggesting a highly conserved inhibitory role (Hatsuzawa et al., 2003; Yizhar et al., 2004; Gracheva et al., 2006; Zhang et al., 2006; Chen et al., 2011; Williams et al., 2011; Li et al., 2019). Null mutations in *C. elegans Tomosyn* (*tom-1)* show a dramatic increase in evoked neurotransmitter release (Gracheva et al., 2006; McEwen et al., 2006; Burdina et al., 2011). Whether this phenotype is caused by reduced decoy SNARE activity remains unclear given the scaffold and SNARE domains are both required in tandem to suppress release (Burdina et al., 2011). Genetic interactions in *C. elegans* suggest release suppression by Tomosyn may prevent Unc13/Unc-18-independent SNARE assembly. The total elimination of release in *unc-13* and *unc-18* single mutants indicate SV priming normally occurs exclusively via an Unc13/Unc18-dependent mechanism. Remarkably, double mutants of *tom-1/unc-13* and *tom-1*/*unc-18* partially restore the loss of evoked and spontaneous release in *unc-13* and *unc-18* single mutants, indicating Tomosyn suppresses a pathway that would otherwise bypass Unc13 and Unc18 to generate dysregulated priming (McEwen et al., 2006; Gracheva et al., 2010; Hu et al., 2013). *In vitro* reconstitution experiments indicate Tomosyn does not interfere with Unc13/Unc18-chaperoned SNARE assembly, suggesting Tomosyn can only engage Syx1 in an Unc13/Unc18-independent manner (Li Y. et al., 2018). NSF disassembly of the Tomosyn/t-SNARE complex leads to Unc18 capture of Syx1 for incorporation into productive SNARE complexes (Hatsuzawa et al., 2003; Li Y. et al., 2018). *In vivo*, *tom-1* enhanced release is exaggerated by the open-Syx1 mutation, causing a further increase in *tom-1* sensitivity to the acetylcholinesterase inhibitor aldicarb (Tien et al., 2020). Enhanced SV fusion in *tom-1* exceeds the residual release in *tom-1/unc-13* and *tom-1/unc-18* double mutants, indicating Tomosyn also suppresses SNARE assembly within the traditional Unc13/Unc18 priming pathway. Together, these data indicate Tomosyn ensures tight regulation of SNARE complex assembly by acting as a failsafe to prevent dysregulated Unc13/Unc18-independent priming of Syx1.

## Conclusion and Future Directions

**FIGURE 7:**
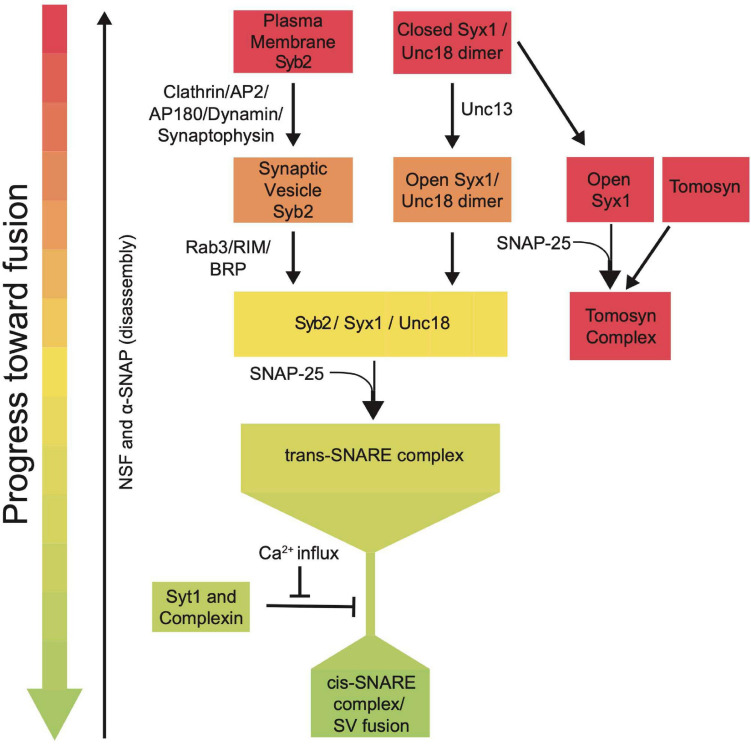
Summary flowchart showing current models for SRP regulation of SNARE complex assembly and SV fusion.

SRPs guide SNARE interactions during multiple steps of the SV fusion cycle by localizing SNARE assembly, regulating Ca^2+^-dependent SNARE zippering, recycling SNAREs post-fusion and inhibiting dysregulated SV priming (Figure 7). Given their critical roles in synaptic communication, it is not surprising that mutations in these genes cause a host of severe human neurological disorders (Melom and Littleton, 2011; Engel et al., 2016; Lipstein et al., 2017; Redler et al., 2017; Salpietro et al., 2019; Abramov et al., 2021; Melland et al., 2021). SRPs and SNAREs are broadly expressed in all neurons and are ideally positioned to regulate intrinsic synaptic release strength and presynaptic plasticity. Given presynaptic output and plasticity mechanisms display wide heterogeneity across neuronal subtypes and at individual release sites, SRPs are likely subject to transcriptional and post-translational control that alters their function. Indeed, SRPs can regulate release differences by controlling SV availability, spontaneous release rate, and SV priming location (Hu et al., 2013; Melom et al., 2013; Cho et al., 2015; Böhme et al., 2016; Fulterer et al., 2018; Aponte-Santiago and Littleton, 2020; Pooryasin et al., 2021).

Several examples of SRP regulation that alter synaptic function or plasticity have been characterized. Phosphorylation of Tomosyn by protein kinase A (PKA) prevents its ability to inhibit SV availability, thereby enhancing SV release to facilitate plasticity and memory formation (Baba et al., 2005; Chen et al., 2011; Ben-Simon et al., 2015). Additionally, PKA phosphorylation of a Drosophila Cpx isoform occurs during activity-dependent retrograde signaling that reduces its clamping function, allowing activity-dependent increases in spontaneous SV release to promote synaptic growth and development (Yoshihara et al., 2005; Huntwork and Littleton, 2007; Barber et al., 2009; Choi et al., 2014; Cho et al., 2015; Andreae and Burrone, 2018). The synaptic signaling molecule nitric oxide (NO) enhances synaptic transmission by modifying Cpx function in Drosophila, with NO-regulated S-nitrosylation of the Cpx C-terminus altering SNARE-binding and enhancing evoked release (Robinson et al., 2018). Transcriptional regulation of Cpx can also modulate synaptic strength in Drosophila, with caloric intake and insulin signaling decreasing synaptic transmission by increasing Cpx levels downstream of the translational inhibitor FOXO (Mahoney et al., 2016). Similarly, the synaptic levels of Unc18 and Syx1 are finely tuned to regulate SV priming dynamics that are required to support presynaptic homeostatic plasticity at Drosophila NMJs (Ortega et al., 2018). Alternative splicing of Unc13 in Drosophila and *C. elegans* results in unique isoforms that alter the protein’s length, allowing the MUN domain to position and control SV priming at varying distances from AZ Ca^2+^ channel clusters (Hu et al., 2013; Böhme et al., 2016; Reddy-Alla et al., 2017; Fulterer et al., 2018; Pooryasin et al., 2021). In Drosophila, newly formed AZs first accumulate the long Unc13B splice variant before the shorter Unc13A variant arrives as AZs mature. Unc13B does not support efficient evoked release but is sufficient for spontaneous fusion, while Unc13A is required for SV priming for evoked but not spontaneous release (Böhme et al., 2016). Short isoforms of Unc13 in *C. elegans* also promote greater evoked release than longer isoforms (Hu et al., 2013), suggesting alternative Unc13 splicing represents a general mechanism for altering the efficiency of SV release by controlling where SV priming occurs along the AZ. These examples highlight only a subset of the mechanisms by which SNARE function might be dynamically controlled by SRPs. Future studies will certainly provide additional insights into how regulation of these essential presynaptic gatekeepers of brain communication contribute to the diversity of synaptic function and plasticity observed across distinct neuronal populations.

## Author Contributions

Both authors wrote and edited the manuscript, contributed to the article, and approved the submitted version.

## Conflict of Interest

The authors declare that the research was conducted in the absence of any commercial or financial relationships that could be construed as a potential conflict of interest.

## Publisher’s Note

All claims expressed in this article are solely those of the authors and do not necessarily represent those of their affiliated organizations, or those of the publisher, the editors and the reviewers. Any product that may be evaluated in this article, or claim that may be made by its manufacturer, is not guaranteed or endorsed by the publisher.
